# Atypical replantation and reconstruction of frozen ear: A case report

**DOI:** 10.1097/MD.0000000000020068

**Published:** 2020-05-15

**Authors:** Zdeněk Dvořák, Igor Stupka

**Affiliations:** a Department of Plastic and Aesthetic Surgery, St. Anneʼs University Hospital; b Faculty of Medicine, Masaryk University, Brno, Czech Republic.

**Keywords:** auricular replantation, ear replantation, freezing cold injury, venous congestion

## Abstract

**Rationale:**

The first successful ear replantation was performed by Pennigton in 1980 in Sydney. At least 84 ear replantations have been described in the literature over a period of 37 years since the first case. The authors have not found any previous case of frozen ear replantation in the literature.

**Patient concerns:**

We report the case of a 38-year-old man, who had an injury to the head while working with a machine.

**Diagnosis:**

The patient suffered total traumatic avulsion of the left ear. The ear was wrapped in moistened, sterile gauze and was transported on dry ice. At the time of admission to our department, the amputated ear was frozen to stiff, solid nonelastic matter.

**Interventions:**

We attempted replantation. Despite repeated arterial thrombosis during surgery, the ear was successfully replanted with arterial and venous anastomosis.

**Outcomes:**

Venous congestion occurred within 9 h of surgery and was treated using leeches. Freezing cold injury developed during reattachment. The radix and proximal parts of the helix exhibited necrosis and so were reconstructed by contralateral conchal cartilage graft, which was wrapped with a local subauricular skin flap. On completion of treatment, a satisfactory shape was achieved, although the replanted and reconstructed left auricle slightly was smaller than the contralateral auricle.

**Lessons learned:**

Our report confirms that the replantation of a frozen, amputated ear is possible, and we suggest that ear replantation should be the method of choice for the treatment of ear loss even under these conditions.

## 1 Introduction

The foundations for ear replantation surgery were based on the work of Buncke in 1966, when he experimented with replantation of the auricle in rabbits.^[1]^ Despite this initial work, the first successful human ear replantation was not performed until 22 years later, by Pennington from Sydney.^[2,3]^ More than 80 cases of ear replantation have been carried out with a reported success rate of about 60%–90%.^[4,5,6]^ However, this rate is not completely representative, because successful replantations are predominantly reported in the literature.^[4]^ Successful auricle replantation has been reported even after 33 hours post-avulsion.^[7]^ In our department of plastic and aesthetic surgery, we have documented 6 successful replantations of pinna.^[8]^ Venous congestion was the most significant postoperative problem.^[4,9,10,11,12]^ Males are the most often affected by loss of ear; with the most common causes being vehicle accident, dog bites, fights, and work-related trauma.

The blood supply of the earlobe originates from the branches of the external carotid artery; that is, the superficial temporal and posterior auricular arteries. The arterial helical arcade provides a connection between the anterior and posterior arterial systems. This arcade is essential to the survival of the auricle after replantation with microvascular anastomosis of a single vessel.^[13,14]^

Protocols relating to the treatment of amputated parts are well known. Proper treatment consists of wrapping the amputated part/s in wet gauze, and placing into a plastic bag within another plastic bag containing 1 part ice and 2 parts water to achieve a cooling temperature of 4°C.^[15,16,17]^ However, errors regularly occur in primary care, the smallest of which is storage of the amputated part without cooling. A more detrimental mistake is to place the amputated parts on dry ice or cover with frozen vegetables, causing the part to be frozen at −18°C. Such frost-damaged tissues are not suitable for replantation.^[18,19]^ The present case study reports an example of this unfortunate situation.

## 2 Case report

**Figure 1:**
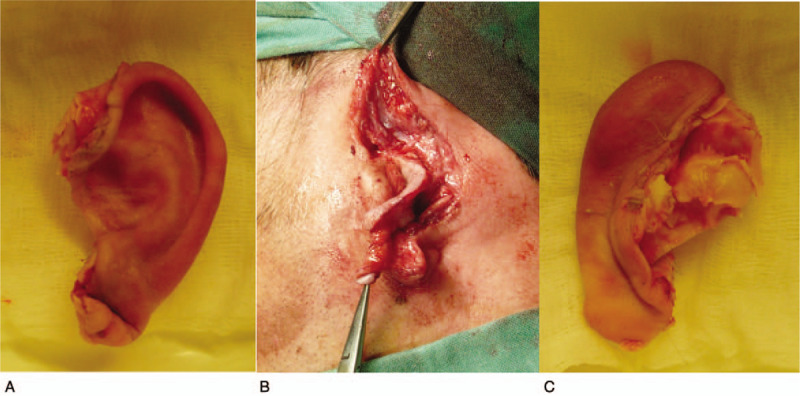
Photographs demonstrating the preoperative status of the amputated left ear: A) viewed from the front, B) defect on the head, and C) viewed from the rear.

Our patient was a 38-year-old, fit and well laborer, heavy smoker up to 20 a day who suffered a head injury by a parquetting machine during his night shift. This caused avulsion of whole left pinna. The crushed and dilacerated left ear was transported by paramedics wrapped in moistened gauze and stored on dry ice, causing the entire ear to freeze in a rigid, non-elastic. The appearance of the amputated ear from the front and back sides, and the defect on the patient's head are shown in Figure 1.

Despite very unfavourable conditions, ear replantation was undertaken. In the operating room under general anaesthesia, debridement of the wound was performed followed by exploration of suitable donor vessels. A median branch of the posterior auricular artery was identified, and end-to-end anastomosis was performed. Prior to release of the arterial clamp, 2500 units of heparin were administered intravenously. The posterior auricular artery thrombotized 3 times despite repeated vascular end-to-end anastomoses resulting in technical inability to perform transient vascular anastomosis. Subsequently, all supply branches were gradually prepared, but none were suitable for anastomosis. As a final option, the earlobe was extensively prepared and a suturable artery was found in the depth of the tissue. An end-to-end anastomosis was created between the posterior auricular and lobular arteries. An operating microscope with 36× magnification and an 11 to 0 microsuture were used to create the anastomosis. A drainage vein was identified and sutured at the dorsal upper edge of the auricle. The left ear was revascularized after 8 hours’ surgery. Anticoagulation therapy was provided for the first 5 days with Pentoxifylline (800 mg) and Heparin (10,000 units) per day; and for the following 10 days with Pentoxifylline (800 mg daily) and Nadroparin (2850 units) twice a day.

Unfortunately, venous congestion developed within 9 hours after surgery. This situation was resolved by regular leeching. The leeches typically exhibited a 4-fold increase in volume after attachment, and would detach without intervention after 20 or 25 minutes. Blood flowed profusely from the site of attachment for another 8 to 10 hours, which reduced the development of venostasis. Leeches were applied at approximately 12 hours intervals. The patient required a total of 7 units of transfused red blood cells during the course of leeching to maintain the haemoglobin level at a minimum of 80 g/dL. In total, 17 leeches were applied.

**Figure 2:**
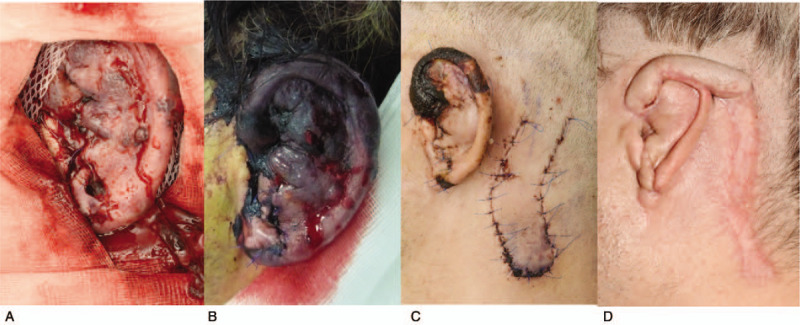
Photographs illustrating the course and complications of reattachment of the left auricle: A) appearance on the second postoperative day, B) appearance on the fifth postoperative day (venostatic crisis), C) appearance of the auricle 7 days after the delay operation, 3 weeks after replantation, and D) appearance of the left ear with a retroauricular flap before removing of a pedicle.

Despite repeated attacks of venostasis (as seen on day 5, Fig. 2B) and the development of bloody blisters on the surface of the auricle, the ear was gradually reattached. Unfortunately, multi-resistant *Pseudomonas aeruginosa* infection occurred at the base of the pinna. After antibiotic treatment, necrosis of the radix and ventral part of the helix was progressively demarcated, as can be seen in Figure 2C.

The resulting necrosis of the root and upper part of the helix was solved by a local retro-auricular flap. Preparatory flap delay was performed when necrosis appeared 14 days after replantation, and the flap was transposed into the defect after 7 days.

The flap, with a Doppler verified central artery (a branch of the posterior retroauricular artery), was elevated and then sutured back. The flap was elevated similarly to the auriculotemporal Washio flap,^[20]^ but more dorsally to avoid disruption of the ear reattachment. Necrectomy of the auricle was performed the following week, and the retro-auricular flap was moved to the defect of the helix. The flap pedicle and the donor site were covered by COM 30 combined dressing fabric (a 3-layer combined covering consisting of a polyester mesh, polyurethane foam and polyamide knitted fabric; made by VUP Medical Brno, Czech Republic). The COM 30 was replaced by a full-thickness skin graft from the supraclavicular area after 2 days. The patient stayed in hospital for 30 days.

The nutritional pedicle from the retro-auricular region was disconnected 6 weeks after replantation (Fig. 2D).

The state of the attached ear was evaluated 9 months after surgery. Compared with the healthy right side, the replanted left auricle was reduced, shrivelled, and retracted into the lower half following debridement and retro-auricular infection. It also exhibited abundant tissue in the root area and at the top of the helix.

**Figure 3:**
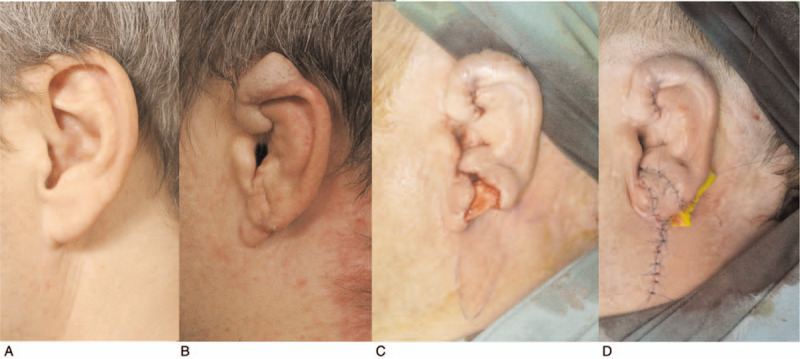
Photographs of reconstruction of the missing part of the lower third of the left auricle: A) image of the healthy right side, mirrored and overturned, B) the affected ear before the final operation, 9 months after replantation, C) appearance after implantation of the cartilaginous graft into the lower third of the auricle, and D) appearance of the ear after completion of the reconstruction operation.

**Figure 4:**
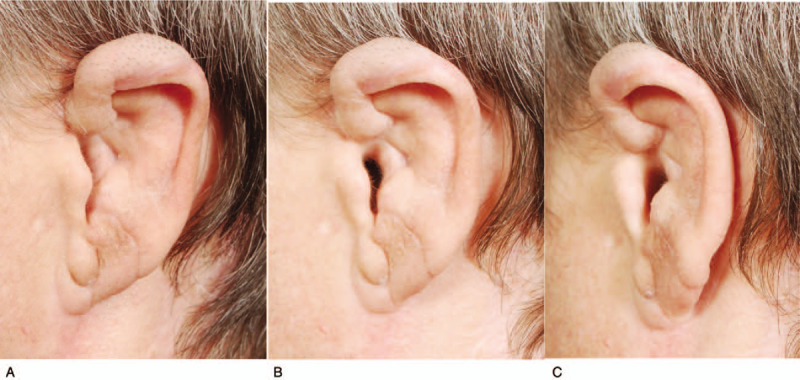
Appearance of the ear 13 months after replantation and reconstruction of the auricle: A) left side view from the front, B) side view, and C) left side view from the back.

We decided to correct the insufficient lower third of the auricle by inserting a cartilaginous graft from the contralateral cavum conchae, and covering with a local flap (Fig. 3A– D). Contralateral conchal cartilage (10 × 25 mm) was harvested and carved to match the remaining cartilage of the helical rim. The cartilage graft was buried and sutured to fit the contour, then covered by transposition of the local subauricular flap. The final result of auricle reconstruction 13 months after replantation is shown in Figure 4.

In the present case, replantation and subsequent reconstruction were successful in achieving a physiological appearance of the auricle despite the initial condition of the amputated tissue. The left ear remained slightly smaller and more tightly inclined to head than on the healthy side, but the patient was satisfied with the result of reconstruction and refused further interventions.

## 3 Discussion

A number of techniques have been developed for reattachment of the amputated auricle, including the composite graft method – or the Mladick technique – involving the removal of skin and insertion into the postauricular subcutaneous pocket.^[21]^ Another technique is Baudet modification, which involves removal of dorsal skin from the auricle, cartilage fenestration, and implantation.^[22]^ However, none of these techniques can achieve equivalent reconstructive results to microvascular ear replantation.^[5,23]^

Jung reported that in 2011, 52 ear replantations had been performed worldwide. After searching Medline and Scopus databases with the keywords “Ear”, “Auric^∗^”, and “Replantation”, we identified an additional 8 records.^[24,25,26,27,28,29,30,31]^ Articles written in English and published between 2011 and 2017 returned a total of 14 reports of ear replantation. In addition, 5 records were found in other languages (Chinese, Turkish, Spanish, and Polish) describing the replantation of an additional 18 auricles.^[32,33,34,35,36]^ In total; up to the end of 2017, at least 84 replantations had been described in the literature in a period of 37 years. The rarity of the procedure is probably due to the rare occurrence of monotrauma of the ear without any additional head or neck injuries. Furthermore, the necessity of a supermicrosurgical procedure, demanding postoperative care resulting from long operating procedures, requirement of venostasis management (commonly through the use of leeches), risk of long-term bleeding with frequent need for blood transfusion and requirement for long-term hospitalisation contribute to the rarity of cases.

Based on the cited literary sources, it appears that the following steps are commonly performed in order to maintain the arterial supply of the auricle: primary repair of the posterior auricular artery, anastomosis of the superficial temporal artery, venous graft, arteriovenous shunt, arterialisation of the venous system,^[8,24]^ or distal radial artery perforator graft.^[37]^

To maintain venous drainage; direct venous anastomosis, venous graft, superficial temporal vein pedicle graft, or arteriovenous shunt are typically used. It is advisable to perform ear replantation even if venous anastomosis cannot be performed because, among the measurable outcomes, only the duration of surgery is significantly different between patients with or without venous anastomosis.^[38]^ The survival rate of the replanted ear is around 68%, regardless of whether venous anastomosis is performed. In practice, this means that when the arterial component is malfunctioning, then microsurgical revision is indicated. In the case of venous component failure, which the most common complication of auricular replantation, treatment of venous congestion is indicated.

Venous congestion occurs in up to 75% of patients.^[4]^ A small incision, leeches, or “chemical leeches” (intra–replant subcutaneous heparin injections) are commonly used for venous decompression. Approximately 50% of replantation patients require blood transfusions due to permanent bleeding.^[4]^

After replantation of frozen amputated tissue, a freeze injury can develop on the surface of the pinna. Locally, the changes appear similar to *Congelatio erythematosa, bullosa et necroticans*. According to Hota and Singh, this could be classified as I, II, or III degree frostbite.^[39]^ According to the literature, local cold irritation leads to reflex changes in circulation and thrombogenesis.^[40]^ It is difficult to assess the degree to which freezing of the auricle or an avulsion-type injury affect the incidence of thrombosis of the vessels during replantation, or whether the severity of cold damage or the distance from the nutritional artery have an effect on the development of radix helix necrosis. In the present study, the results of cold injury of the pinna were healed or fully expressed 3 weeks after replantation. The patient reported no pain or chronic hypersensitivity of the replanted auricle, which is common in the case of frostbite injuries. The patient did, however, complain of cold intolerance. The authors have found no other reference case of replantation of frozen auricle.

The best functional and aesthetic results following ear loss can be achieved by auricular replantation; despite the long, personally and materially demanding surgery and long-term hospitalisation. The efforts of the surgical team during pinna replantation should be focused on reconstruction of the arterial and venous vessels (to minimise venostasis and blood loss) and ensuring the immediate availability of leeches and blood units to cover postoperative blood loss. Postoperative care is difficult mainly due to the management of venostasis, which is the most common complication of ear replantation.

The presented case demonstrates that it is possible to achieve satisfactory ear reattachment, even if the auricle is damaged by freezing. However, it is necessary to consider the complications resulting from simultaneous cold injury and subsequent loss of tissue that can be solved by corrective operations similar to auricle reconstruction.

This case also demonstrates the resistance of auricular tissue to cold, crushing, and venostasis during reattachment. Therefore, auricular replantation should always be the primary treatment for ear loss. Other methods should be considered only when replantation is not possible.

## Author contributions

**Conceptualization:** Zdenek Dvorak, Igor Stupka.

**Data curation:** Zdenek Dvorak.

**Formal analysis:** Igor Stupka.

**Investigation:** Zdenek Dvorak.

**Methodology:** Zdenek Dvorak, Igor Stupka.

**Project administration:** Zdenek Dvorak.

**Supervision:** Igor Stupka.

**Validation:** Zdenek Dvorak.

**Writing – original draft:** Zdenek Dvorak.

**Writing – review & editing:** Zdenek Dvorak.
